# Ropivacaine-loaded hydrogels for prolonged relief of chemotherapy-induced peripheral neuropathic pain and potentiated chemotherapy

**DOI:** 10.1186/s12951-023-02230-5

**Published:** 2023-12-02

**Authors:** Xin Qing, Renbin Dou, Peng Wang, Mengni Zhou, Chenchen Cao, Huiwen Zhang, Gaolin Qiu, Zhilai Yang, Jiqian Zhang, Hu Liu, Shasha Zhu, Xuesheng Liu

**Affiliations:** 1 Key Laboratory of Anesthesia and Perioperative Medicine of Anhui Higher Education Institutes, Department of Anesthesiology, The First Affiliated Hospital of Anhui Medical University, Anhui Medical University, Hefei, 230032 China; 2 Department of Obstetrics and Gynecology, Reproductive Medicine Center, The First Affiliated Hospital of Anhui Medical University, Hefei, 230032 China

**Keywords:** Chemotherapy, Chemotherapy-induced peripheral neuropathic pain, Local anesthetic, MHC-I, Hydrogel

## Abstract

Chemotherapy can cause severe pain for patients, but there are currently no satisfactory methods of pain relief. Enhancing the efficacy of chemotherapy to reduce the side effects of high-dose chemotherapeutic drugs remains a major challenge. Moreover, the treatment of chemotherapy-induced peripheral neuropathic pain (CIPNP) is separate from chemotherapy in the clinical setting, causing inconvenience to cancer patients. In view of the many obstacles mentioned above, we developed a strategy to incorporate local anesthetic (LA) into a cisplatin-loaded PF127 hydrogel for painless potentiated chemotherapy. We found that multiple administrations of cisplatin-loaded PF127 hydrogels (PFC) evoked severe CIPNP, which correlated with increased pERK-positive neurons in the dorsal root ganglion (DRG). However, incorporating ropivacaine into the PFC relieved PFC-induced CIPNP for more than ten hours and decreased the number of pERK-positive neurons in the DRG. Moreover, incorporating ropivacaine into the PFC for chemotherapy is found to upregulate major histocompatibility complex class I (MHC-I) expression in tumor cells and promote the infiltration of cytotoxic T lymphocytes (CD8^+^ T cells) in tumors, thereby potentiating chemotherapy efficacy. This study proposes that LA can be used as an immunemodulator to enhance the effectiveness of chemotherapy, providing new ideas for painless cancer treatment.

**Supplementary Information:**

The online version contains supplementary material available at 10.1186/s12951-023-02230-5.

## Introduction

Although chemotherapy is an effective method for treating solid tumors, nearly 80% of the patients experience chemotherapy-induced peripheral neuropathic pain (CIPNP) in the therapeutic process [1]. Due to patient intolerance, CIPNP can lead to detrimental dose modifications and premature withdrawal from chemotherapy [2]. Additionally, the long-term effects of CIPNP can have a profound impact on the quality of life and survival of patients [3]. In the clinical setting, gabapentin, antidepressants, and opioids are commonly used to relieve CIPNP; however, their analgesic effects are unsatisfactory and often lead to many side effects [4]. Compared to the aforementioned analgesics, local anesthetics (LAs) have many potential advantages, including effectiveness, low cost, and relatively few side effects, making them widely used in analgesic management [5–7]. However, currently available LAs cannot entirely fulfill the demand for long-lasting anesthesia and high doses result in irreversible toxicity [8, 9]. In order to overcome these deficiencies, Chen et al. developed an injectable electrospun fiber-hydrogel composite loaded with clonidine and ropivacaine for long-term walking analgesia [10]. Zhao et al. designed hydrogel microneedles to deliver lidocaine hydrochloride and enhance local long-lasting analgesia [11]. Peng et al. demonstrated that liposomes loaded with ropivacaine exhibited a longer duration of local anesthesia without obvious toxicity [12]. However, there are few studies on using local anesthetics to relieve CIPNP.

Conventional systemic chemotherapy is the most prevalent approach for treating tumors and preventing recurrence [13]. However, chemotherapy drugs through intravenous administration faces the challenge of overcoming transport barriers before effectively reaching the cancer site, resulting in only a small fraction of the drug being delivered to the tumor. Moreover, higher systemic doses can give rise to undesirable side effects in normal tissues [14]. Intratumoral chemotherapy (ITC) has emerged as a new adjuvant treatment that is used prior to irradiation or surgery owing to several potential advantages, including assured precision in the local delivery of drugs, complete perfusion of drugs within and around the lesion, high drug intratumoral concentration, and rapid eradication of the tumor burden [15–17]. Thus, ITC has wider clinical applications in most solid tumors [18]. However, the high clearance limits the application of ITC [19]. Therefore, various systems, such as hydrogels [20], nano/microparticles [21, 22], micelles [23, 24], and liposomes [25] have been investigated for localized drug delivery to prolong high intratumoral drug concentrations.

High-dose chemotherapy drugs are often used in clinics to improve the therapeutic effects on tumors [26]. Although it is possible to improve clinical outcomes in some certain cases, the administration of high-dose chemotherapy may cause dose-limited adverse effects, including neurotoxicity, bone marrow suppression, gastrointestinal toxicity, cardiotoxicity, as well as nausea and vomiting [27, 28]. Therefore, improving the efficacy of chemotherapeutic drugs without increasing their dose is another challenge. Interestingly, in addition to relieving pain, LAs have shown antitumor potential. There have been several studies indicating that LAs can kill tumor cells [29], inhibit tumor metastasis [30], prevent postoperative recurrence [31] and enhance the effectiveness of conventional anti-tumor treatments [32]. However, it is not clear whether LAs can benefit the treatment of tumors by modulating the immune system. Dysfunctional antigen presentation caused by defects in major histocompatibility complex class I (MHC-I) is a common mechanism for immune evasion by tumor cell [33, 34]. Keisuke et al. have found that inhibiting autophagy upregulates MHC-I in tumor cells, leading to improved antigen presentation, potentiated anti-tumor T cell response, and inhibited tumor growth [35]. Furthermore, our previous research showed that the local anesthetic, ropivacaine, inhibited autophagy by weakening lysosomal degradation [36]. Therefore, ropivacaine may increase MHC-I levels in tumor cells, which would enhance the efficacy of chemotherapy by mobilizing the immune system.

**Fig. 1:**
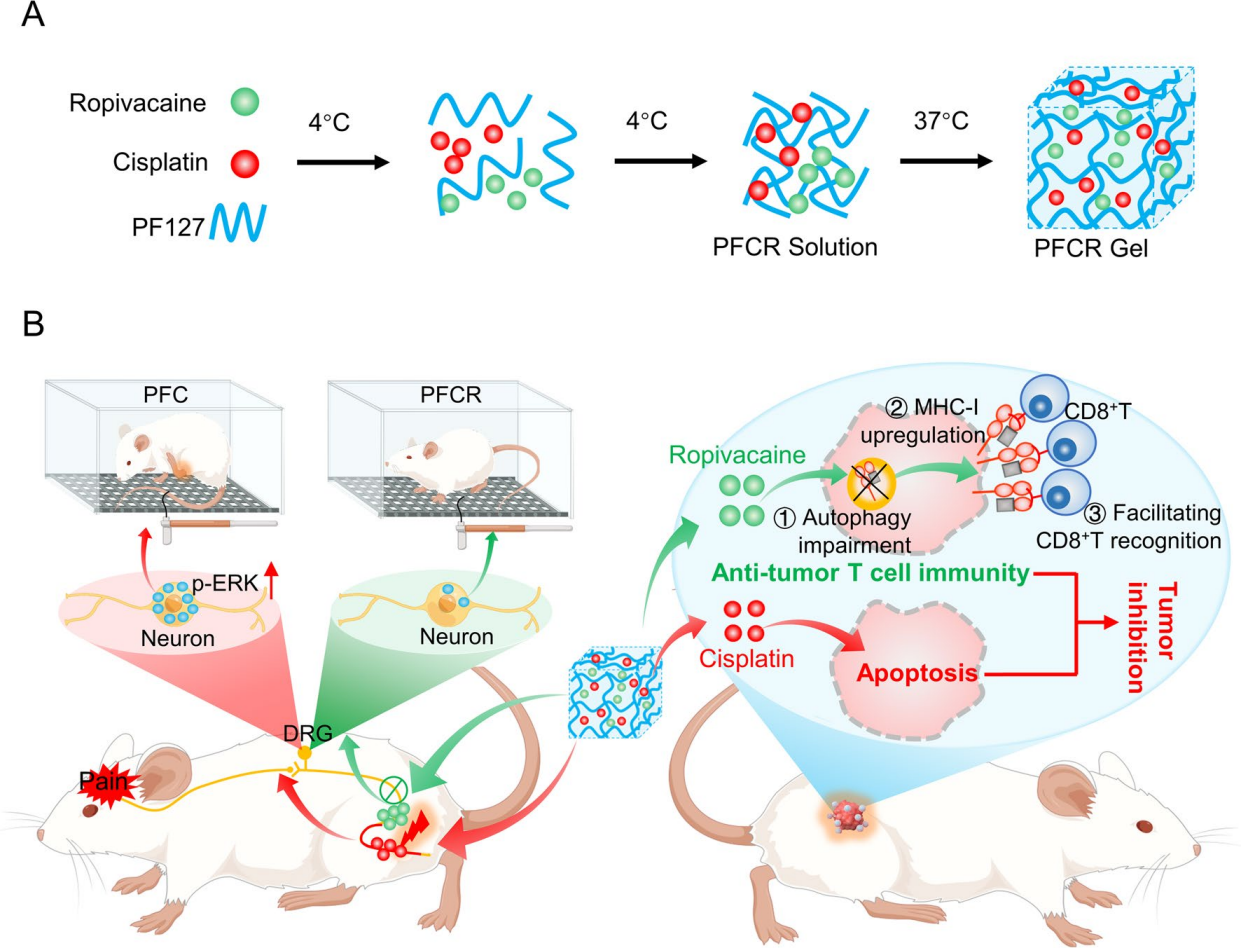
A schematic diagram showing that a PF127 hydrogel loaded with cisplatin and ropivacaine (PFCR) relieves chemotherapy-induced peripheral neuropathic pain (CIPNP) and potentiates chemotherapy efficacy by enhancing T cell immunity. **A** Preparation of PFCR hydrogel. **B** Mechanism of PFCR in relieving CIPNP and anti-tumor. Ropivacaine released from PFCR blocks the transmission of pain signal and reduces the number of pERK positive nerons in DRG, alleviating CIPNP. Cisplatin released from PFCR directly kills tumors, while ropivacaine enhances the expression of MHC-I in tumor cells, facilitates their recognition by CD8^+^ T cells, which further kills tumor cells

Pluronic F127 (PF) is a thermo-responsive biocompatible polymer approved by the Food and Drug Administration (FDA) [37]. In this study, we prepared PF127 hydrogels loaded with cisplatin and LA ropivacaine (PFCR) for painless in situ chemotherapy. We found that multiple administrations of cisplatin-loaded PF127 hydrogels (PFC) evoked severe CIPNP, which correlated with increased pERK-positive neurons in the dorsal root ganglion (DRG). However, incorporating ropivacaine into the PFC relieved PFC-induced CIPNP for more than ten hours and reduced the number of pERK-positive neurons in the DRG. Moreover, incorporating ropivacaine into the PFC for chemotherapy upregulated MHC-I expression in tumor cells and promoted the recognition of tumor cells by cytotoxic T lymphocytes (CD8^+^ T cells), thereby potentiating chemotherapy efficacy. This is the first study to show that the introduction of LA into chemotherapeutic agents synergistically relieves CIPNP and potentiates chemotherapy (Fig. 1). In addition, this study innovatively proposes that LA can be used as an immunemodulator to enhance the effectiveness of chemotherapy, providing new ideas for painless cancer treatment.

## Materials and methods

### Antibodies and agents

Anti-p62 (ab109012), anti-LC3B antibody (ab192890), anti-MHC class I (ab281901), Alexa Fluor 568 (ab175473), and Alexa Fluor 488 (ab150077) were purchased from Abcam. Anti-phospho-p44/42 MAPK (Erk1/2) (4703s) was purchased from Cell Signaling Technology. Anti-mouse CD16/32-TruStain FcX™ (101320), anti-mouse CD8a-APC (100711), anti-mouse CD45-PE-cy7 (103114) antibodies, and one-step TUNEL In Situ Apoptosis Kit (Green, FITC) (E-CK-A320) were purchased from Elabscience. The enhanced chemiluminescence (ECL) kit was purchased from Biological Industries. Pluronic F127 (P2443) was purchased from Sigma-Aldrich. Ropivacaine (R413090) and cisplatin (P4394) were obtained from Mackline.

### Fabrication and characterization of F127 hydrogels

PF127 hydrogels were prepared according to a “cold method” described previously [37]. 25% PF127 solution (PF) was prepared by dissolving 0.25 g Pluronic® F127 into 1 mL of sterile deionized water and stirring at 4 °C until completely dissolved. To prepare the PFC hydrogel, 2.5 mg of cisplatin was mixed with 1 mL of PF127 solution and stirred gently at 4 °C until a hydrogel was formed. To prepare the PFR hydrogel, 10 mg of ropivacaine was mixed with 1 mL of PF127 solution and stirred gently at 4 °C until a hydrogel was formed. To prepare the PFCR hydrogel, 2.5 mg of cisplatin and 10 mg of ropivacaine were added to 1 mL of PF127 solution and mixed gently at 4 °C to obtain a hydrogel. The surface morphology of the hydrogels was analysed using scanning electron microscopy (SEM; JSM-6330F, JEOL, Japan).

### Rheological characterization

Rheological characterization of the hydrogels was performed using a strain-controlled shear rheometer (MCR 302; Anton-Paar, Austria). The rheological properties of hydrogel, storage modulus (G′), and loss modulus (G′′) were measured at a strain of 1% and a frequency of 1.0 Hz as the temperature was increased from 4 to 40 °C at a rate of 1 °C/min. The frequency dependence, shear behavior were measured at 37 °C. All measurements were performed using a 40 mm parallel plate geometry with a 0.6 mm gap size.

### In vitro drug release study

A glass bottle containing PFCR was placed in an incubator at 37 °C. When the solution changed from liquid to gel, 4 mL of sterile deionized water (pH = 7.4) was slowly added to the surface and placed in a shaker at a temperature of 37 °C and a speed of 70 rpm. At time points (0 h, 2 h, 4 h, 6 h, 8 h, 10 h, 12 h, 24 h, 36 h, 48 h), 1 mL of the solution was collected and then an equal volume of deionized water was added [38]. Ropivacaine and cisplatin concentrations were determined by a Micro UV–Vis Spectrophotometer (LIFEREAL, FC-1100) and ICP-MS (ELAN DRC II, PerkinElmer, Waltham), respectively.

### Western blotting

RIPA lysis buffer was used to extract proteins from 4T1 cells, while a phosphatase-protease cocktail inhibitor (P1050, Beyotime, China) was added to the buffer. The resulting supernatant was boiled for 10 min. Protein concentration was determined using a BCA Protein Assay Kit (BB-3401, Bestbio, China). Equal amounts of protein were loaded onto 13.5% sodium dodecyl sulfate-polyacrylamide gels for electrophoresis, and then transferred to nitrocellulose membranes. Subsequently, the membranes were blocked with 5% skim milk at room temperature for 2 h, followed by overnight incubation with primary antibodies at 4 °C. After washing off the primary antibodies, the membranes were incubated with horseradish peroxidase-conjugated secondary antibodies for 2 h. The bands were detected using a chemiluminescence (ECL) detection reagent kit and visualized using a chemiluminescence instrument (Amersham Imager 600; GE Healthcare, Japan).

### Immunofluorescence

Mice were anesthetized with pentobarbital and subjected to intracardial perfusion with PBS, followed by perfusion with 4% paraformaldehyde. The dorsal root ganglion of L4–L5 or tumor tissues underwent transfer to 4% paraformaldehyde for a duration of 4 to 6 h. Subsequently, tissues was placed in 30% sucrose at 4 °C overnight. The tissues were then embedded in OCT compound (SaKura TissueTek, USA) and subsequently sliced into 10 μm sections using a cryostat (MNT, SLEE, Germany). Once the frozen sections were washed with PBS, they were blocked with 3% bovine serum albumin (BSA) at a temperature of 37 °C. The slides were then subjected to an overnight incubation with a primary antibody at 4 °C, followed by one hour incubation with a fluorescent secondary antibody at 37 °C. After staining with DAPI and a subsequent PBS wash, the slides were observed using a Zeiss LSM800 confocal microscope. And pERK-positive cells were counted using Image J software with “Image/Adjust/Color threshold/Yen Thresholding method” function. Each group included a statistical analysis of six tissue slices from three mice per group.

### Cell culture

The 4T1 mouse breast cancer cell line was acquired from the China Center for Type Culture Collection, located in Wuhan, Hubei, China. These cells were cultured at 37 °C in a humidified atmosphere containing 5% CO_2_ in Dulbecco’s modified Eagle’s medium (DMEM) supplemented with 10% fetal bovine serum (FBS), 100 U/mL of penicillin, and 100 µg/mL of streptomycin.

### Animals

Eight-week-old female BALB/c mice were purchased from Shanghai SLAC Laboratory Animal Co., Ltd. (Shanghai, China). The mice were housed in a controlled environment with a 12-h light–dark cycle, constant room temperature, and humidity. They were allowed to acclimate to the experimental setting for 1 week, during which they had free access to food and water. The housing and care of the mice followed the guidelines outlined in the Guide for Care and Use of Laboratory Animals of the National Institutes of Health. The Ethics Committee of Anhui Medical University approved all animal procedures, and efforts were taken to minimize the use of animals in this study.

### Anti-tumor assessment

Mice were anesthetized using sodium pentobarbital. In the immediate vicinity of the trochanter, a total of 1 × 10^5^ 4T1 cells in 100 mL of sterile PBS were injected into the muscular tissue near the nerve [39]. Six days after inoculation, the tumor diameters reached 4–6 mm [40] and the mice were randomly divided into four groups and injected with 100 µL of PF, PFC or PFCR in situ every 3 days for 14 days. Tumor volume was calculated using the following formula: length × width^2^/2 = tumor volume (mm^3^). The tumor size was measured every 2 days [41].

### Flow cytometry analysis

The 4T1 cells were plated in 24-well plates at a density of 5 × 10^4^ cells per well. After incubating in DMEM for 12 h, the cells were co-cultured with PF, PFR, PFC, or PFCR (40 µL added to 500 µL of the culture medium) for 8 h. Following a quick PBS wash, the cells were collected and stained with an APC anti-MHC-I antibody. Subsequently, the percentage of MHC-I^+^ cells was determined using a BD FACSVerse flow cytometer and the data were analyzed using FlowJo software from TreeStar.

The tumor-bearing mice were treated in the same manner as described above. Once the treatment was completed, the tumors were collected and cut into small pieces. These pieces were then placed in a solution of PBS, collagenase type IV (1 mg/mL), and DNase I (40 µg/mL). The mixture was incubated for 45 min at 37 °C with shaking at 100 rpm. For flow cytometric analysis, the cell suspensions were first treated with anti-CD16/32 to block Fc receptors. They were then stained with PE/cyanine7 anti-CD45 and APC anti-CD8 antibodies for 30 min on ice. The percentage of CD45^+^CD8^+^ T cells was determined using a BD FACSVerse flow cytometer. The resulting data was analyzed with the FlowJo software (TreeStar).

### Behavior assessment

Mice were anesthetized using sodium pentobarbital. A total of 1 × 10^5^ 4T1 cells in 100 µL of sterile PBS were injected into the muscular tissue in the immediate vicinity of the nerve near the trochanter, immediately distal to where the posterior biceps semitendinosus branches off the common sciatic nerve [39]. Untreated mice or tumor-inoculated mice were randomly divided into four groups (PF, PFC, PFC + Rop, PFCR) and the hydrogels were injected in the same location of the tumor. Mechanical withdrawal thresholds were examined at 0, 4, 10, and 24 h after the last administration. To assess the mechanical withdrawal thresholds, a 2450 series electronic von Frey aesthesiometer (IITC 2091) was used. Before testing, the mice were placed in plastic cages (5 cm × 5 cm × 8 cm) that had wire-net floors. They were allowed to acclimatize for 1 h. Place the rigid tip of the electronic Von Frey sensor on the plantar surface of the hind paw, and press it slowly until the withdrawal reflex was observed. The force that triggered the withdrawal reflex was recorded. The test was repeated three times and the test interval was 5 min, then the average mechanical withdrawal threshold was calculated.

### Assessment of systemic toxicity

The systemic toxicity of the hydrogels was further evaluated. We recorded the weight changes in all mice immediately after tumor implantation. H&E staining of the main organs was conducted to assess any potential adverse effects of the hydrogels. Blood samples were collected to evaluate a complete blood panel analysis and blood biochemistry tests [42].

### Statistical analysis

The statistical results were presented by mean ± standard deviation (SD). The comparison between the two sets of data was analyzed using a Two-Tailed Student’s t-test. The comparison between three or more sets of data was analyzed using One-Way or Two-Way repeated measures analysis of variance with Tukey’s post hoc test. *p < 0.05, **p < 0.01, and ***p < 0.001 were considered statistically significant. Statistical analysis was carried out using GraphPad Prism 7 Software.

## Results

### Characterization of PFCR

**Fig. 2:**
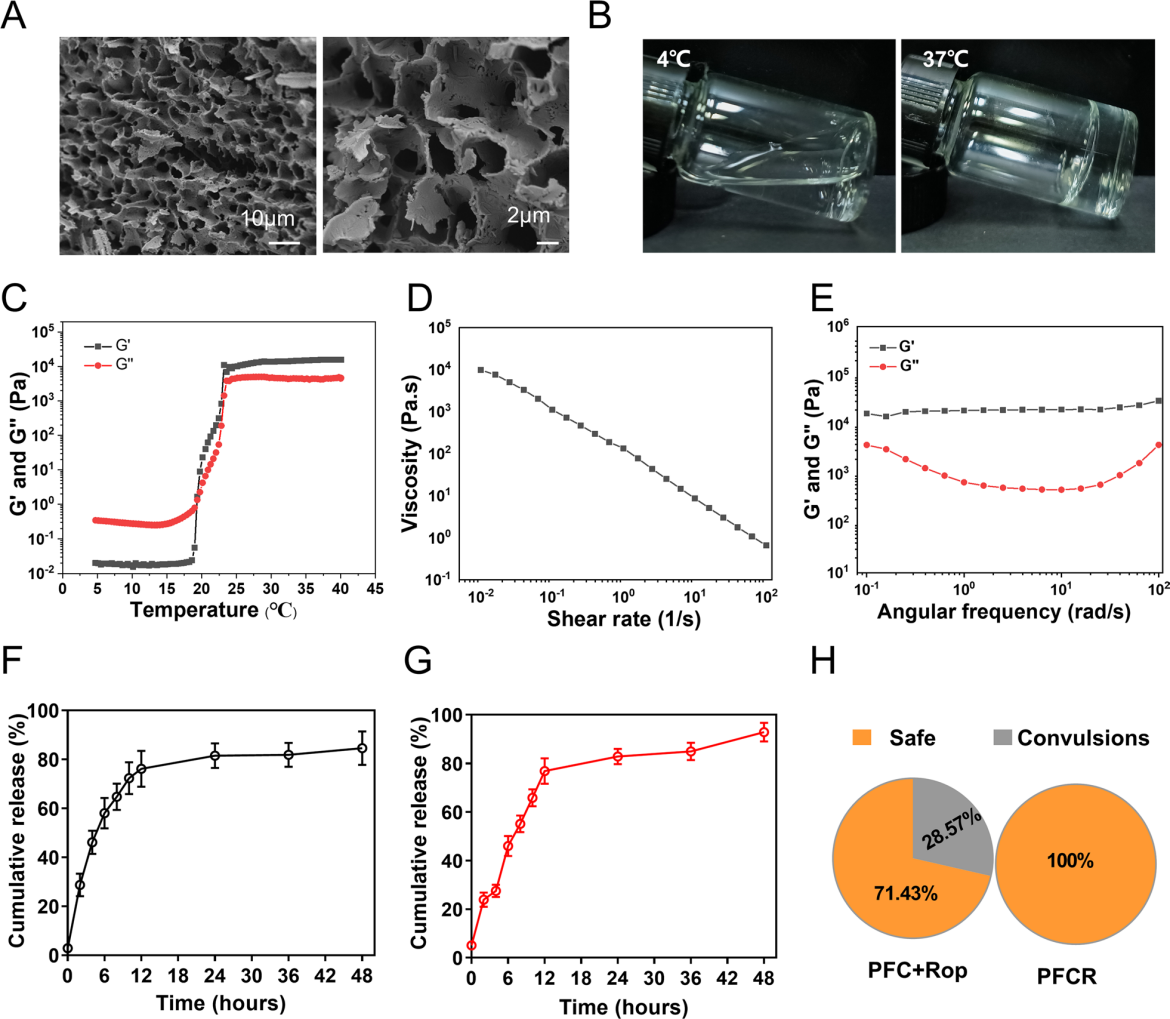
Characterization and properties of PFCR. **A** Scanning electron microscopy (SEM) images of PFCR hydrogel revealed its structure and morphology. **B** The fluidity of the PFCR hydrogel at both 4 and 37 °C was investigated, indicating its temperature-dependent behavior. **C** Rheology studies showed the temperature-dependent viscoelastic properties of PFCR aqueous dispersion. **D** Steady-shear rheology demonstrated the shear-thinning behavior of PFCR. **E** Frequency-dependent rheology analysis of PFCR hydrogel at 37 °C. **F** In vitro cumulative release experiments of ropivacaine from PFCR (n = 3). **G** In vitro cumulative release experiments of cisplatin from PFCR (n = 3). **H** The occurrence of convulsions in mice administered with PFCR or a cisplatin-loaded PF127 hydrogel plus free ropivacaine (PFC + Rop) was statistically analyzed (n = 7). Data are presented as the mean ± SD. PFC + Rop: cisplatin-loaded PF127 hydrogel plus free ropivacaine; PFCR: cisplatin and ropivacaine-coloaded PF127 hydrogel

Conventional cisplatin chemotherapy causes CIPNP and systemic toxicity. To overcome these limitations, we synthesized PFCR as an implant for painless and potentiated intratumoral chemotherapy. As observed using SEM, the PFCR showed a porous structure (Fig. 2A). In addition, the PFCR solution is a free-flowing liquid at 4 °C that transforms into a hydrogel at 37 °C, which facilitates implantation in vivo (Fig. 2B). Then the rheological properties of PFCR were characterized. As shown in Fig. 2C, storage modulus (G′) and loss modulus (G′′) increased with temperature. When the temperature is lower than 19.37 °C, G′′ > G′, indicating liquid-state behavior; when the temperature higher than 19.37 °C, G′′ < G′, suggesting a typical characteristic of the solid-like behavior (Fig. 2C). As the shear rate changed, the viscosity of the hydrogels decreased, indicating a typical shear-thinning behavior (Fig. 2D). Additionally, at 37 °C, G′ exceeded G′′ across the entire frequency range, indicating the presence of typical viscoelastic behavior and the stability of the formed hydrogel system (Fig. 2E). Notably, PFC showed rheological properties similar to those of PFCR (Additional file 1: Fig. S1). The strain sweep of the hydrogel showed the loss modulus (G″) surpassed the storage modulus (G′) when the strain exceeded ~ 5%, suggesting that the transition from the gel state to the solution state (Additional file 1: Fig. S2A). As shown in the creep test, at 4 °C, the hydrogel exhibited weaker compressive strength, susceptibility to creep, and a lack of viscoelastic recovery properties (Additional file 1: Fig. S2B). Conversely, at 37 °C, the hydrogel exhibited greater compressive strength, reduced susceptibility to creep, and strong elastic recovery properties (Additional file 1: Fig. S2C). The results of swelling experiment showed that the hydrogel swelled after absorbing water, reaching equilibrium within approximately 90 min. Moreover, the swelling rate of the hydrogel was approximately 195% (Additional file 1: Fig. S3). Additionally, we investigated the release of ropivacaine and cisplatin from PFCR. According to the release profile, approximately 80% of ropivacaine and 90% of cisplatin were ultimately released, reaching a release plateau around 24 h later (Fig. 2F, G). The release amount at an acid condition (pH 6.0) of ropivacaine and cisplatin was similar to that at a neutral condition (Additional file 1: Fig. S4A, B). In addition, high concentrations of ropivacaine in circulation can lead to convulsions and even death in mice. Therefore, we tested the biosafety of PFCR after intratumoral injection. As shown in Fig. 2H, PFCR injection did not induce convulsions, while 28.57% of free ropivacaine-injected mice exhibited convulsions. These results suggest that PFCR is biosafe and may be used for the prolonged relief of CIPNP.

### Incorporating ropivacaine into hydrogels prolonged relief of CIPNP

**Fig. 3:**
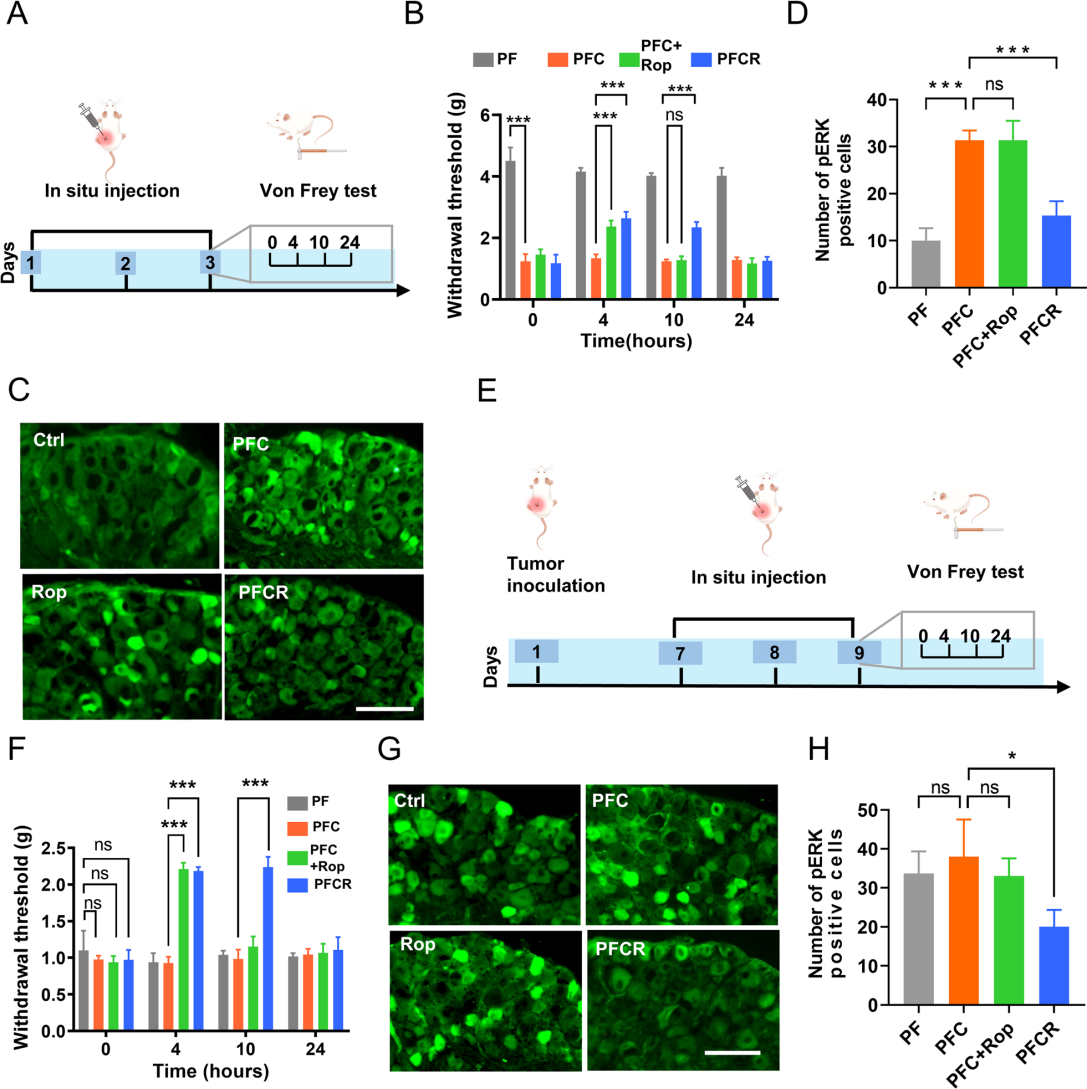
Incorporation of ropivacaine into hydrogels prolongs the relief of CIPNP. **A** Schematic diagram of drug administration for chemotherapy in mice. **B** Mechanical withdrawal threshold in mice was tested at 0, 4, 10, and 24 h after treatment with different hydrogels (n = 6). **C** p-ERK immunofluorescent staining in the dorsal root ganglion (DRG) of mice at 10 h after the last administration, scale bar = 100 μm. **D** Statistical results of pERK positive neurons in DRG (n = 6 slices from 3 mice per group). **E** Schematic diagram of drug administration for chemotherapy in tumor-inoculated mice. **F** Mechanical withdrawal threshold in tumor-inoculated mice was tested at 0, 4, 10, and 24 h after treatment with different hydrogels (n = 6). **G** p-ERK immunofluorescent staining in DRG of tumor-inoculated mice at 10 h after the last administration. scale bar = 100 μm. **H** Statistical results of pERK positive neurons in DRG (n = 6 slices from 3 mice per group). Data are presented as the mean ± SD. *p < 0.05, **p < 0.01, ***p < 0.001. PF: PF127 hydrogel; PFC: cisplatin-loaded PF127 hydrogel; PFC + Rop: cisplatin-loaded PF127 hydrogel plus free ropivacaine; PFCR: cisplatin and ropivacaine-coloaded PF127 hydrogel

Effective relief from CIPNP is a major challenge in chemotherapy. In addition, CIPNP treatment is separate from chemotherapy in the clinic, which brings inconvenience to cancer patients. Therefore, in this study, we investigated whether the incorporation of ropivacaine into chemotherapeutic agents could relieve CIPNP. PFC, PFCR, and free ropivacaine plus PFC (PFC + Rop) were injected near the sciatic nerve for 3 consecutive days, and the pain in mice was assessed by recording a mechanical stimulus-induced withdrawal response using an electronic von Frey apparatus (Fig. 3A) [43]. The baseline paw withdrawal threshold (PWT) of mice was determined before the last injection. The results showed that the PWT of mice in the PFC, PFC + Rop, and PFCR groups was significantly reduced compared to that in the control group (Fig. 3B), indicating that CIPNP was induced by cisplatin. However, after the last injection, the PWT of mice in the PFC + Rop and PFCR groups significantly increased for more than 4 and 10 h, respectively (Fig. 3B). These results suggested that PFCR administration effectively prolonged the duration of CIPNP relief. To substantiate these results, phosphorylated ERK (pERK)-positive cells of the dorsal root ganglion (DRG) were assessed. DRG is where the cell bodies of primary sensory neurons are located. ERK1/2 is a classic mitogen-activated protein kinase (MAPK), the activated form of ERK1/2, p-ERK1/2, stimulates the generation and release of inflammatory factors and amplifies pain signals [44, 45]. As shown in Fig. 3C, D, at 10 h after the last treatment, the number of pERK positive cells in DRG of the PFC and PFC + Rop group were increased significantly; however, this was remarkably reversed in PFCR-treated mice, confirming the prolonged relief of CIPNP by PFCR.

The approach of incorporating ropivacaine into cisplatin-loaded hydrogels was further evaluated in a mouse model of cancer pain. 4T1 tumors were inoculated near the sciatic nerve to induce spontaneous cancer pain [39]. To determine tumor location, we conducted a thorough gross anatomical examination of the tumor and the adjacent neural structures, the sciatic nerve was embedded in the tumor mass with the tumor tissue completely surrounding the nerve (Additional file 1: Fig. S5A, B). And cisplatin-loaded hydrogels were injected into the tumor near the sciatic nerve to induce CIPNP (Fig. 3E). Interestingly, the subsequent cisplatin-loaded hydrogel treatment did not further aggravate the cancer pain (Additional file 1: Fig. S6A), which was probably due to the fact that the pain induced by cancer was already severe enough to reach a “glass ceiling”. However, compared to the control and PFC treatments, the PFC + Rop treatment significantly increased the PWT of mice for at least 4 h, whereas the PFCR treatment significantly increased the PWT for more than 10 h (Fig. 3F). The number of pERK-positive cells in the DRG was then determined. As shown in Fig. 3G, H, PFCR treatment remarkably reduced the number of pERK-positive cells in the DRG at 10 h, confirming the relief of pain. These results suggest that additional administration of free ropivacaine could temporarily relieve CIPNP, while doping ropivacaine into PF127 hydrogels could prolong relief of pain induced by chemotherapeutic drugs in normal mice and cancer pain mice. Notably, incorporating ropivacaine into PF127 hydrogels also relieved spontaneous cancer pain in mice (Additional file 1: Fig. S6B).

### Incorporation of ropivacaine into hydrogels increases MHC-I in vitro

**Fig. 4:**
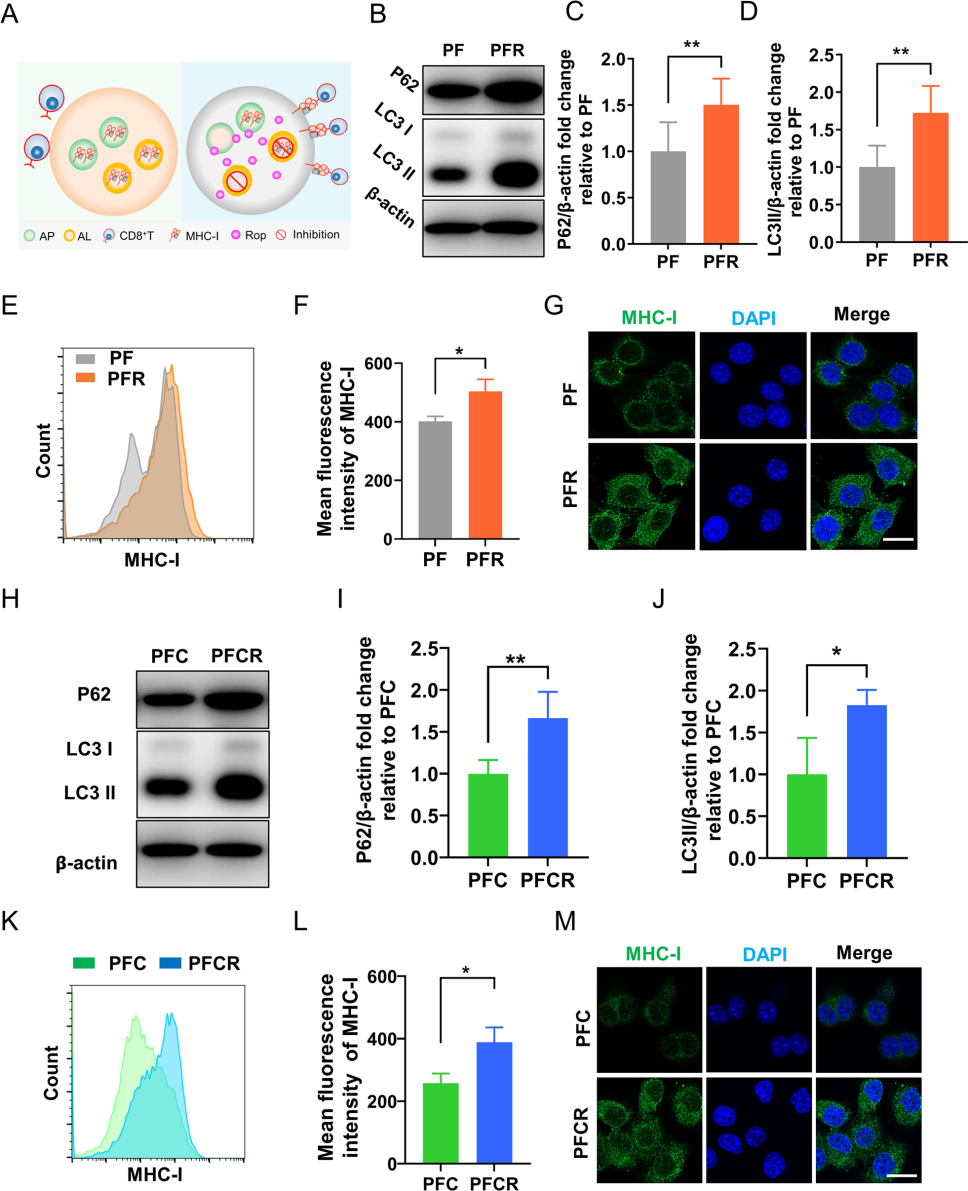
Incorporation of ropivacaine into hydrogels increases MHC-I in vitro. **A** Schematic diagram of upregulation of MHC-I by ropivacaine. *AP* autophagosome, *Rop* ropivacaine, *AL* autolysosome. **B** Western blot results of LC3II and P62 in 4T1 cells treated with PF and PFR for 8 h, respectively. **C**, **D** Statistical results of western blots of LC3II and P62 (n = 4). **E**, **F** The mean fluorescence intensity (MFI) of MHC-I in 4T1 cells treated with PF and PFR for 8 h was detected by flow cytometry (n = 4). **G** MHC-I immunofluorescent staining. Scale bar = 20 μm. **H** Western blot results of LC3II and P62 in 4T1 cells treated with PFC and PFCR for 8 h, respectively. **I**, **J** Statistical results of western blots of LC3II and P62 (n = 4). **K**, **L** The mean fluorescence intensity (MFI) of MHC-I in 4T1 cells treated with PFC and PFCR for 8 h was detected by flow cytometry (n = 4). **M** MHC-I immunofluorescent staining. Scale bar = 20 μm. Data are presented as the mean ± SD. *p < 0.05, **p < 0.01, ***p < 0.001. *PF* PF127 hydrogel, *PFC* cisplatin-loaded PF127 hydrogel, *PFR* ropivacaine-loaded PF127 hydrogel, *PFCR* cisplatin and ropivacaine-coloaded PF127 hydrogel

Despite their analgesic applications, the anti-tumor effectiveness of LAs remains unclear. For instance, whether LAs can mobilize the immune system to promote tumor chemotherapy remains to be elucidated. Impaired antigen presentation caused by MHC-I deficiency is a common mechanism of immune evasion by tumor cells [46]. Keisuke and his colleagues found that the degradation of MHC-I was depended on autophagy. Impairment of autophagy restores MHC-I, improves antigen presentation, potentiates anti-tumor T cell responses, and suppresses tumor growth [35]. Our previous research showed that ropivacaine damages autophagy [36]. Therefore, we hypothesized that the inhibiting autophagy with ropivacaine would lead to the upregulation of MHC-I expression (Fig. 4A). To verify our hypothesis, we tested the effect of ropivacaine-induced autophagy impairment. As shown in Fig. 4B, D, PFR treatment significantly increased LC3II and P62 levels compared to PF treatment, demonstrating autophagy impairment. Furthermore, the results of flow cytometry showed that the MFI of MHC-I in PFR-treated cells was significantly higher than that in PF-treated cells (Fig. 4E, F). In addition, we observed that the fluorescence signals for MHC-I in PFR-treated cells were stronger than those in PF-treated cells via confocal microscopy (Fig. 4G). These results demonstrate that ropivacaine-loaded hydrogels upregulated MHC-I levels in cells.

Next, we tested whether ropivacaine could upregulate MHC-I levels in cisplatin-treated cells. As shown in Fig. 4H, J, PFCR treatment rather than PFC treatment, increased the levels of LC3II and P62, demonstrating the impairment of autophagy. Furthermore, as shown in Fig. 4K, L, the MFI of MHC-I was significantly higher in PFCR-treated cells than in PFC-treated cells. In addition, we observed stronger MHC-I fluorescence signals in PFCR-treated cells than in PFC-treated cells (Fig. 4M). These results demonstrate that ropivacaine also upregulates MHC-I levels in cisplatin-treated cells, suggesting that ropivacaine could potentiate chemotherapeutic efficacy by enhancing T cell immunity.

### Incorporation of ropivacaine into hydrogels potentiates chemotherapy efficacy by enhancing T cell immunity

**Fig. 5:**
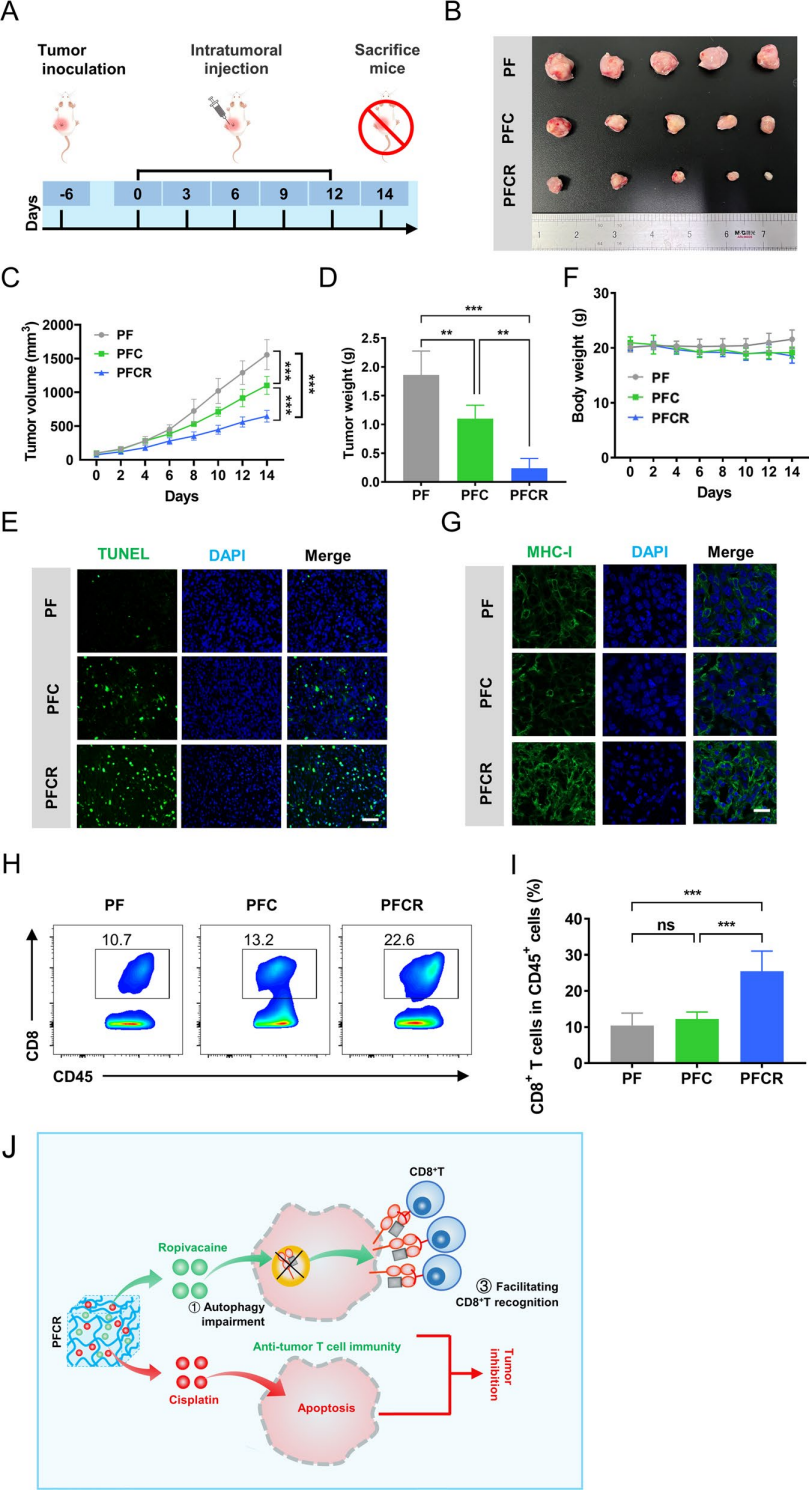
Incorporation of ropivacaine into hydrogels potentiates chemotherapy efficacy by enhancing T cell immunity. **A** Schematic diagram of drug administration for treating the tumors. **B** Tumors after 14 days of different treatments. **C** Average tumor growth curves for 14 days (n = 5). **D** The weight of tumors on the 14th day (n = 5). **E** TUNEL staining for tumor sections was performed to identify apoptotic cells, while DAPI was used to stain the nuclei. Scale bar = 50 μm. **F** Body weight changes during the 14 days of treatments (n = 5). **G** MHC-I immunofluorescent staining in tumors. DAPI was used to stain the nuclei, Scale bar = 20 μm. **H**, **I** Flow cytometry analysis and statistical results of the percent of CD8^+^ T cells in CD45^+^ cells in the tumors (n = 4). **J** Mechanism diagram of PFCR killing tumors. Data are presented as the mean ± SD. *p < 0.05, **p < 0.01, ***p < 0.001. *PF* PF127 hydrogel, *PFC* cisplatin-loaded PF127 hydrogel, *PFCR* cisplatin and ropivacaine-coloaded PF127 hydrogel

We further investigated whether the incorporation of ropivacaine into the cisplatin-loaded hydrogel could potentiate chemotherapeutic effect by enhancing T cell immunity. Tumor-bearing mice were administered with hydrogels every 3 days for 14 days (Fig. 5A). At the end of treatment, PFCR-treated mice showed the smallest tumors among all groups (Fig. 5B). In addition, chemotherapy with PFC exhibited mild anti-tumor effects, while chemotherapy with PFCR exerted strong anti-tumor effects (Fig. 5C, D). There were no statistically significant differences in the anti-tumor effects between the PFR-treated group and the PF-treated group (Additional file 1: Fig. S7A–C). In addition, tumor growth in PFCR-treated mice was significantly slower than that in PF or PFC-treated mice, suggesting that the incorporation of ropivacaine into the hydrogel potentiates the chemotherapeutic effect of cisplatin. Furthermore, the TUNEL assay showed more cell apoptosis in PFCR-treated mice compared to that in PFC- or PF-treated mice (Fig. 5E). Compared with PF group, there was no obvious apoptosis in PFR group (Additional file 1: Fig. S7D). Notably, there were no significant differences in the body weight (Fig. 5F, Additional file 1: Fig. S7E), complete blood panel, function and physiological structure of the major organs of the mice in the different treatment groups (Additional file 1: Fig. S8), which indicated that PFCR had a good biosafety profile.

Next, we investigated the mechanism by which ropivacaine enhanced the chemotherapeutic effects of cisplatin. The killing effect of CD8^+^ T cells on tumor cells is important for the body to remove tumor cells. MHC-I on tumor regulates the specific recognition of tumor cells by CD8^+^ T cells. Therefore, we detected MHC-I expression in mouse tumors. Consistently with the results in vitro, PFCR treatment upregulated MHC-I in mouse tumors (Fig. 5G). Consequently, the infiltration of CD8^+^ T cells into the tumor was significantly increased in PFCR-treated mice compared to that in PF or PFC-treated mice (Fig. 5H, I), revealing that the potentiated chemotherapeutic effect of ropivacaine was achieved by enhancing T cell immunity. Collectively, these results indicated that PFCR chemotherapy demonstrated potent anti-tumor effects. The underlying mechanism is that cisplatin released from PFCR induces tumor cell apoptosis, while ropivacaine upregulates MHC-I in tumor cells and promotes the recognition of tumor cells by CD8^+^ T cells, which further kills tumor cells (Fig. 5J).

## Discussion

Chemotherapy is an effective method for treating solid tumors [47]. CIPNP is a severe adverse effect, which is both distressing and debilitating [1]. CIPNP often causes unbearable pain in patients receiving chemotherapy, which leads to shortening or premature termination of the chemotherapy course and seriously affects the efficacy of chemotherapy [2, 48]. Therefore, it is urgent to develop a chemotherapy strategy that can relieve CIPNP without affecting the chemotherapy effect. In the current study, we designed a PF127 hydrogel loaded with cisplatin and ropivacaine for intratumoral chemotherapy and found that PFCR relieved chemotherapy-induced pain and prolonged analgesia for more than 10 h. In addition, PFCR inhibited autophagy and upregulated MHC-I and T-cell immunity, thus enhancing the tumor-killing effect of cisplatin.

Cisplatin was the first platinum-based compound to receive FDA approval for the treatment of various cancers [49]. Nowadays there are two major problems associated with the clinical use of cisplatin: drug resistance and toxicity [50, 51]. Besides, the particularity of the tumor microenvironment (TME) seriously limits the effectiveness of tumor therapy [52]. In this study, the PF127 hydrogel combined with ITC reduced the systemic toxicity of cisplatin and increased the drug concentration at tumor sites. Hydrogels have 3D networks of cross-linked hydrophilic polymer chains and are widely used because of their numerous advantages [53]. First, it is commonly administered through peri-tumoral and intratumoral injection. This approach allows for increased drug concentration at the tumor sites while reducing the risk of systemic toxicity. Second, the preparation of the drug is a straightforward process that involves mixing hydrogels with other drugs; crosslinking agents, organic solvents, and complicated chemical synthesis steps are not required [54]. Ouyang et al. constructed a novel multifunctional adhesion hydrogel possessed biological safety, satisfactory degradation rate, anti-inflammatory, antibacterial and hemostatic properties for wound hemostasis [42]. Tang et al. prepared a carboxymethyl chitosan (CMCS)/poly-γ-glutamic acid (γ-PGA)/platelet-rich plasma (PRP) hydrogel (CP-PRP hydrogel) for rapid hemostasis and wound healing [55]. Our results showed that doping ropivacaine into PF127 hydrogels could prolong the pain relief induced by chemotherapeutic drugs in both normal and cancer pain mice. Our results suggest that ERK activation in the DRG leads to chemotherapy-induced mechanical hyperalgesia. Previous research has demonstrated that platinum derivatives can affect various MAPKs including ERK, p38, and JNK. The MAPKs activation leads to the production of pronociceptive mediators through different mechanisms, ultimately leading to enhanced and prolonged pain [56]. And studies have demonstrated that cisplatin-induced neuropathic pain is primarily attributed to mitochondrial damage resulting from mDNA-Pt adduct formation, erroneous mitochondrial protein synthesis, reactive oxygen species (ROS) generation [3, 57, 58]. Notably, ROS play an important role in activating the ERK, which in turn contributes to the mitochondrial damage [59, 60]. Our current study further supports these findings, demonstrating that cisplatin treatment induces ERK activation in the DRG, which is involved in pain generation. This suggests that ROS produced as a result of cisplatin treatment may trigger ERK activation, subsequently leading to mitochondrial damage and neuropathic pain. Additionally, Wan et al. revealed that an increase in pERK via the calcineurin/NFAT signaling pathway in the DRG is involved in oxaliplatin-induced CIPNP [61]. Furthermore, Toyoaki et al. found that oxaliplatin-induced pERK causes neuropathic pain [62]. Phosphorylated ERK induces abnormal Na^+^ channel currents, resulting in neuropathy [63]. These findings and our results show that pERK plays a major role in the mechanism of platinum-induced CIPNP in the peripheral nervous system. However, we did not explore whether pERK mediated CIPNP through the central nervous system. Previous studies have shown that ERK activation in the glial cells and neurons of the spinal cord induces neuropathic pain [56]. Activation of ERK in spinal microglia leads to oxaliplatin-induced pain during chemotherapy [64]. The phosphorylation ERK/STAT1 signaling pathway in spinal microglia contributes to bone cancer pain [65]. Therefore, the hydrogel loaded with ropivacaine relieved cancer-related pain likely by inhibiting ERK activation in the central nervous system.

Our recent study showed that a hydrogel co-loaded with the TLR7 agonists imiquimod and ropivacaine increased the infiltration of CD8^+^T cells into the residual tumor tissues to prevent tumor recurrence [66]. Moreover, we designed a PF127 hydrogel doped with ropivacaine, indocyanine green (ICG), and imiquimod for painless enhanced photothermal therapy effect [67]. In this study, we found that a PF127 hydrogel loaded with ropivacaine and cisplatin upregulated MHC-I in tumors by destroying autophagy and increasing the infiltration of CD8^+^T cells into the tumors, thus increasing the anti-tumor effect. We revealed that ropivacaine potentiated chemotherapeutic effects by enhancing T-cell immunity. Notably, unlike previous studies, in this study, we only incorporated LA into the cisplatin-loaded hydrogel without additional immunomodulatory agents, such as imiquimod, for painless tumor treatment, resulting in a satisfactory anti-tumor effect. These results suggest that a combination of local anesthetics and chemotherapeutic agents is an excellent strategy for tumor treatment.

## Conclusion

To summarize, both normal and tumor-bearing mice experienced severe pain when undergoing chemotherapy using PFC, which correlated with an increase in pERK-positive neurons in the DRG. However, incorporating ropivacaine into the PFC relieved PFC-induced pain for more than 10 h and reduced the number of pERK-positive neurons in the DRG. Moreover, incorporating ropivacaine into the PFC for chemotherapy upregulated MHC-I expression in tumor cells and promoted their recognition by cytotoxic T lymphocytes (CD8^+^ T cells), thereby potentiating chemotherapy efficacy. This study suggests that using local anesthesia-based approaches for analgesics can enhance the outcomes of chemotherapy.

### Supplementary Information

**Additional file 1.** **Figure S1**. Characterization and properties of PF127 hydrogel loaded with cisplatin (PFC). (A) Temperature-dependent rheology of PFC aqueous dispersion. (B) The shear-thinning behavior of PFC by steady-shear rheology. (C) Frequency-dependent rheology of PFC hydrogel at 37 °C. **Figure S2**. Characterization and properties of PF127 hydrogel. (A) The strain sweep of the PF127 hydrogel at 37 °C. (B) Creep test of PF127 hydrogel at 4 °C. (C) Creep test of PF127 hydrogel at 37 °C. **Figure S3**. The swelling curve of PF127 hydrogel, n = 3. **Figure S4**. (A) In vitro cumulative release experiments of ropivacaine in an acidic environment (pH = 6.0), n = 3. (B) In vitro cumulative release experiments of cisplatin in an acidic environment (pH = 6.0), n = 3. **Figure S5**. Anatomical location of tumor. (A) Anatomical location of tumor in mice. (B) H&E staining image of tumor side. **Figure S6**. (A) Mechanical withdrawal threshold in tumor-free mice (Ctrl), tumor-inoculated mice and tumor-inoculated mice treated with PFC, n = 7. (B) Mechanical withdrawal threshold in tumor-inoculated mice was measured at 0, 4, 10, and 24 h after different treatments, n = 6. Ctrl: Saline; PF: PF127 hydrogel; Rop: ropivacaine; PFR: ropivacaine loaded PF127 hydrogel. **Figure S7**. Anti-tumor effect of ropivacaine-loaded PF127 hydrogel. (A) Tumors after 14 days of different treatments. (B) Average tumor growth curves for 14 days, n = 5. (C) The weight of tumors on the 14th day, n = 5. (D) TUNEL staining for tumor sections was performed to identify apoptotic cells, while DAPI was used to stain the nuclei. Scale bar = 50 μm. (E) Body weight changes during the 14 days of treatments, n = 5. **Figure S8**. Assessment of systemic toxicity of PF127 hydrogel loaded with cisplatin and ropivacaine (PFCR) in mice. (A) The serum levels of WBC, RBC, Gran, HCT, HGB, MCV, Mon, PLT, MCH, MCHC, AST, ALB, BUN, UREA, CREA in mice teated with different hydrogels for 14 days, n = 4. (B) H&E staining images of the main organs of in mice teated with different hydrogels for 14 days, scale bar = 1mm.

## Data Availability

All data generated and analyzed during this research are included in this published article and its additional file.
