# Supplementary material for: Ropivacaine-loaded hydrogels for prolonged relief of chemotherapy-induced peripheral neuropathic pain and potentiated chemotherapy
Source: J Nanobiotechnology. 2023 Dec 2;21:462. doi: 10.1186/s12951-023-02230-5 (PMC10693114; doi:10.1186/s12951-023-02230-5)
Supplement: Supplementary file 1 — Additional file 1. Figure S1. Characterization and properties of PF127 hydrogel loaded with cisplatin (PFC). (A) Temperature-dependent rheology of PFC aqueous dispersion. (B) The shear-thinning behavior of PFC by steady-shear rheology. (C) Frequency-dependent rheology of PFC hydrogel at 37 °C. Figure S2. Characterization and properties of PF127 hydrogel. (A) The strain sweep of the PF127 hydrogel at 37 °C. (B) Creep test of PF127 hydrogel at 4 °C. (C) Creep test of PF127 hydrogel at 37 °C. Figure S3. The swelling curve of PF127 hydrogel, n = 3. Figure S4. (A) In vitro cumulative release experiments of ropivacaine in an acidic environment (pH = 6.0), n = 3. (B) In vitro cumulative release experiments of cisplatin in an acidic environment (pH = 6.0), n = 3. Figure S5. Anatomical location of tumor. (A) Anatomical location of tumor in mice. (B) H&E staining image of tumor side. Figure S6. (A) Mechanical withdrawal threshold in tumor-free mice (Ctrl), tumor-inoculated mice and tumor-inoculated mice treated with PFC, n = 7. (B) Mechanical withdrawal threshold in tumor-inoculated mice was measured at 0, 4, 10, and 24 h after different treatments, n = 6. Ctrl: Saline; PF: PF127 hydrogel; Rop: ropivacaine; PFR: ropivacaine loaded PF127 hydrogel. Figure S7. Anti-tumor effect of ropivacaine-loaded PF127 hydrogel. (A) Tumors after 14 days of different treatments. (B) Average tumor growth curves for 14 days, n = 5. (C) The weight of tumors on the 14th day, n = 5. (D) TUNEL staining for tumor sections was performed to identify apoptotic cells, while DAPI was used to stain the nuclei. Scale bar = 50 μm. (E) Body weight changes during the 14 days of treatments, n = 5. Figure S8. Assessment of systemic toxicity of PF127 hydrogel loaded with cisplatin and ropivacaine (PFCR) in mice. (A) The serum levels of WBC, RBC, Gran, HCT, HGB, MCV, Mon, PLT, MCH, MCHC, AST, ALB, BUN, UREA, CREA in mice teated with different hydrogels for 14 days, n = 4. (B) H&E staining images of the main organs [file 12951_2023_2230_MOESM1_ESM.docx]

**Ropivacaine-loaded hydrogels for prolonged relief of chemotherapy-induced peripheral neuropathic pain and potentiated chemotherapy**

**Supplementary material**

**Methods**

**Characterization**

For the rheological characterization of PF127 hydrogel, an HR10 rheometer (TA instrument) with a flat stainless steel plate geometry (20 mm diameter) was used and the operating gap distance was 1000 µm. To evaluate the resistance to compression of PF127 hydrogel under 4 °C and 37 °C, creep experiments were performed under a constant stress of 5 Pa for 2 min, followed by measuring strain upon stress removal for 3 min. And strain sweep were measured at 37 °C with the fixed frequency setting at 1 Hz and the strain scanning range setting at 0.1-100%.

**Swelling ratio**

The swelling property of the hydrogel was investigated by immersing sample into PBS (pH = 7.4) at 37°C until reaching a swelling equilibrium state. The swelling ratio (SR) of the hydrogel was calculated as follows: SR (%) = (W_t_ – W_0_)/ W_0_ *100. W_0_ is the initial weight before immersion, W_t_ is the weight of hydrogel after soaking for predetermined time points and carefully wiping off the excess surface water. All tests were carried out in triplicate.

**In vitro drug release study**

In order to simulate tumor microenvironment, drug release was tested in an acidic buffer (pH = 6.0).

**Anti-tumor effect of ropivacaine-loaded PF127 hydrogel**

Mice were anesthetized using sodium pentobarbital. In the immediate vicinity of the trochanter, a total of 1 × 10^5^ 4T1 cells in 100 mL of sterile PBS were injected into the muscular tissue near the nerve. Six days after inoculation, the tumor diameters reached 4 - 6 mm and the mice were randomly divided into two groups and injected with 100 μL of PF, PFR in situ every three days for 14 days. Tumor volume was calculated using the following formula: length × width^2^ / 2 = tumor volume (mm^3^). The tumor size was measured every two days.

**Results**

**Figure S1**

**
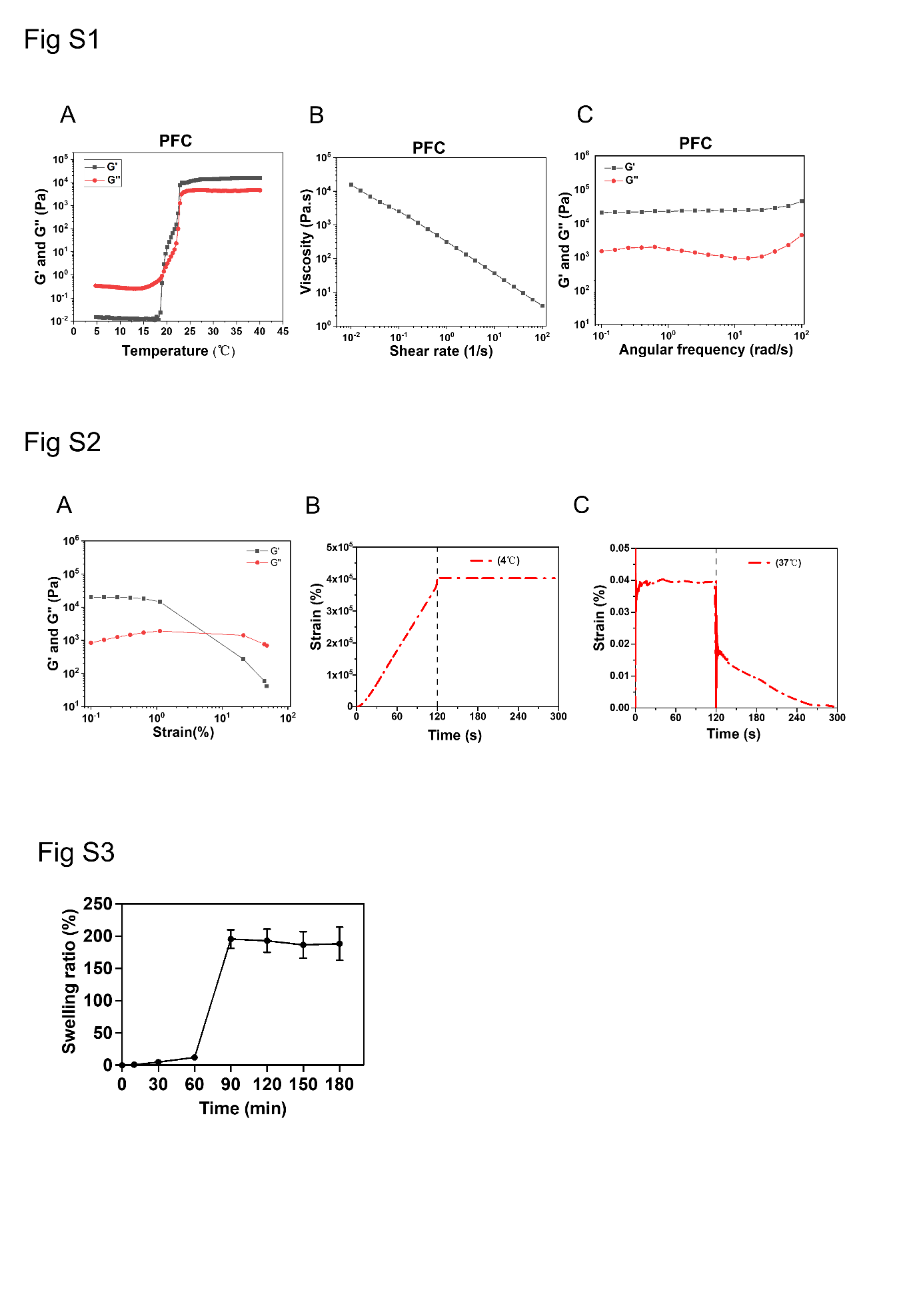
**

Figure S1. Characterization and properties of PF127 hydrogel loaded with cisplatin (PFC). (A) Temperature-dependent rheology of PFC aqueous dispersion. (B) The shear-thinning behavior of PFC by steady-shear rheology. (C) Frequency-dependent rheology of PFC hydrogel at 37 °C.

**Figure S2**


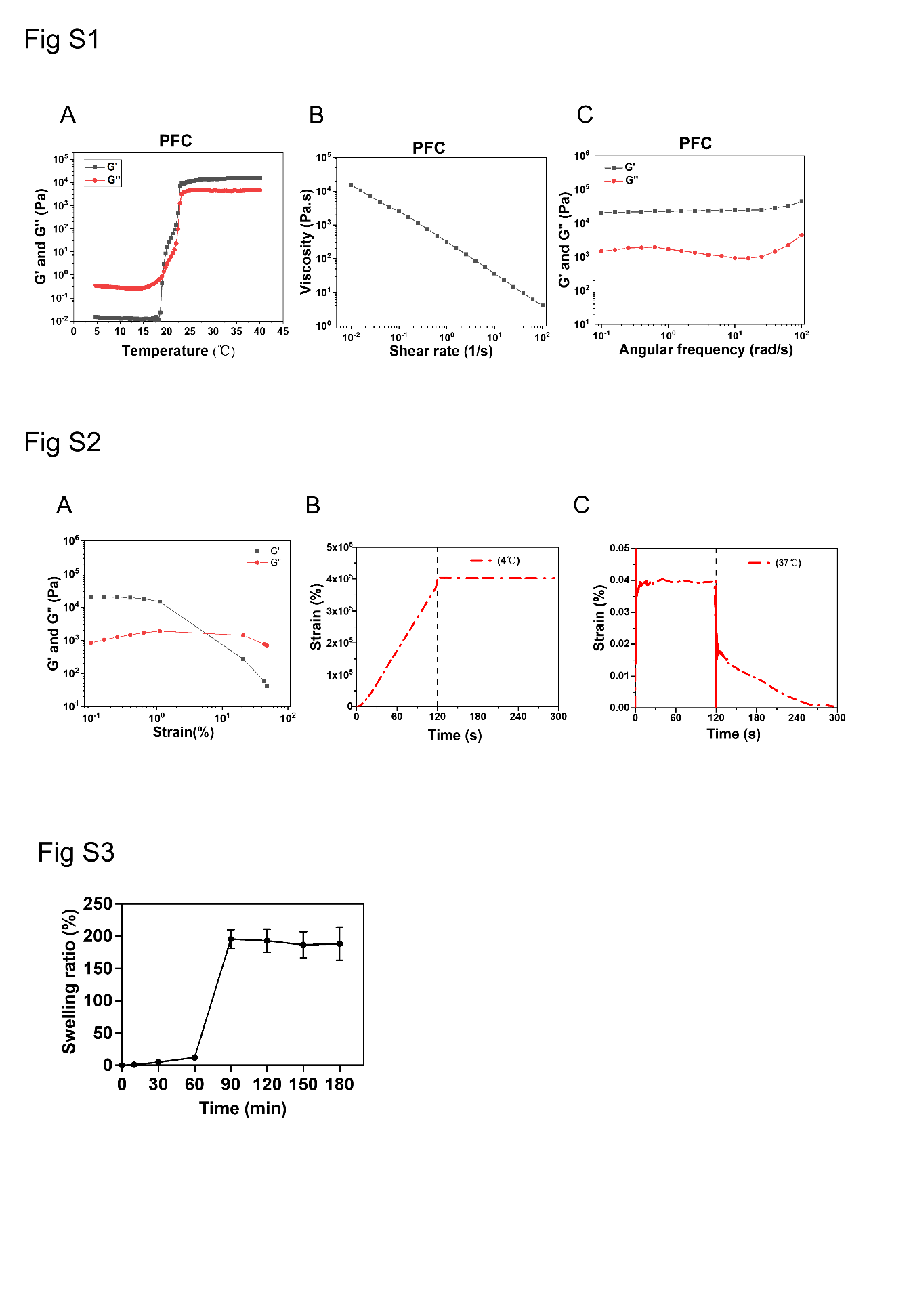


Figure S2. Characterization and properties of PF127 hydrogel. (A) The strain sweep of the PF127 hydrogel at 37 °C. (B) Creep test of PF127 hydrogel at 4 °C. (C) Creep test of PF127 hydrogel at 37 °C.

**Figure S3**


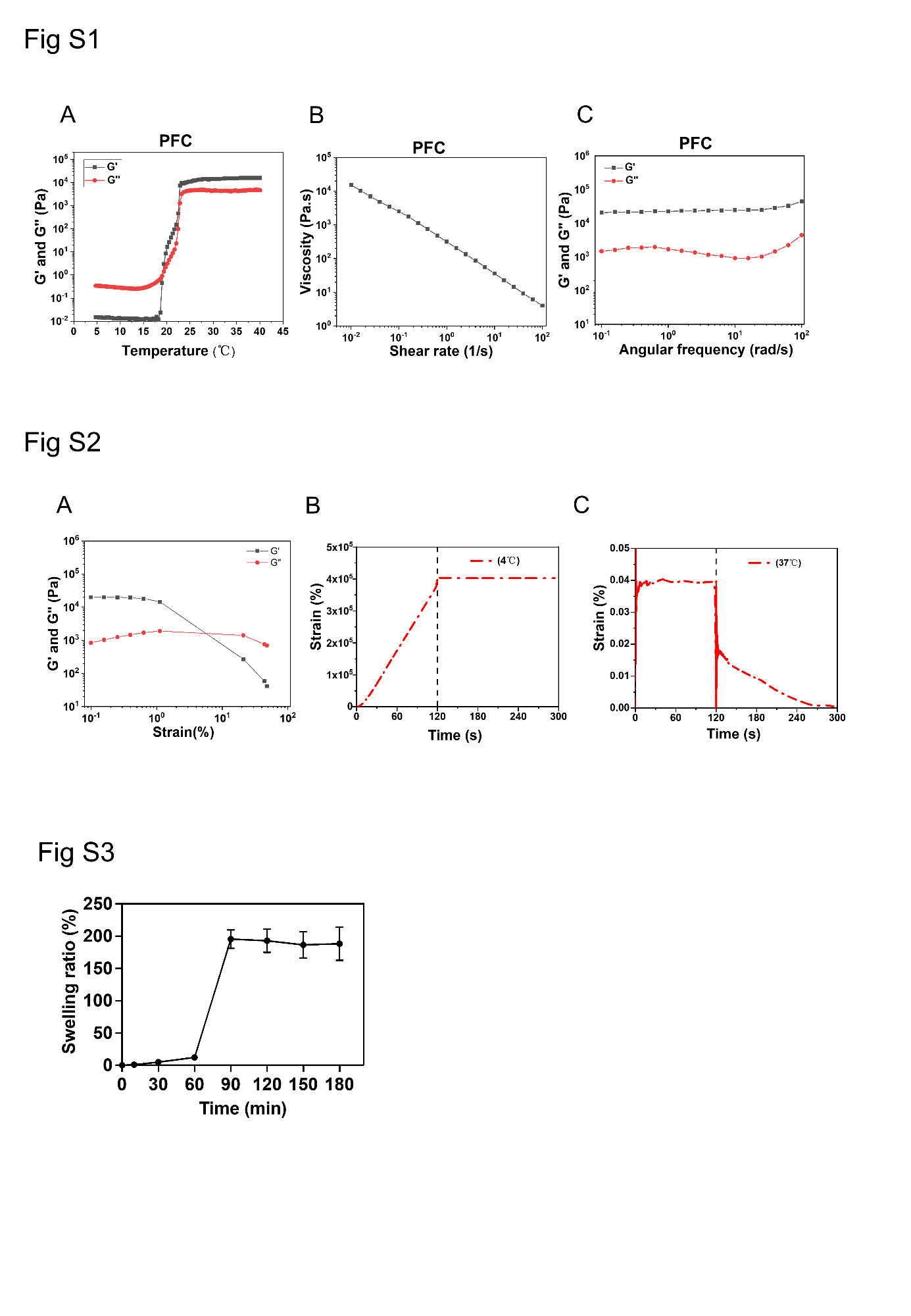


Figure S3. The swelling curve of PF127 hydrogel, n = 3

**Figure S4**


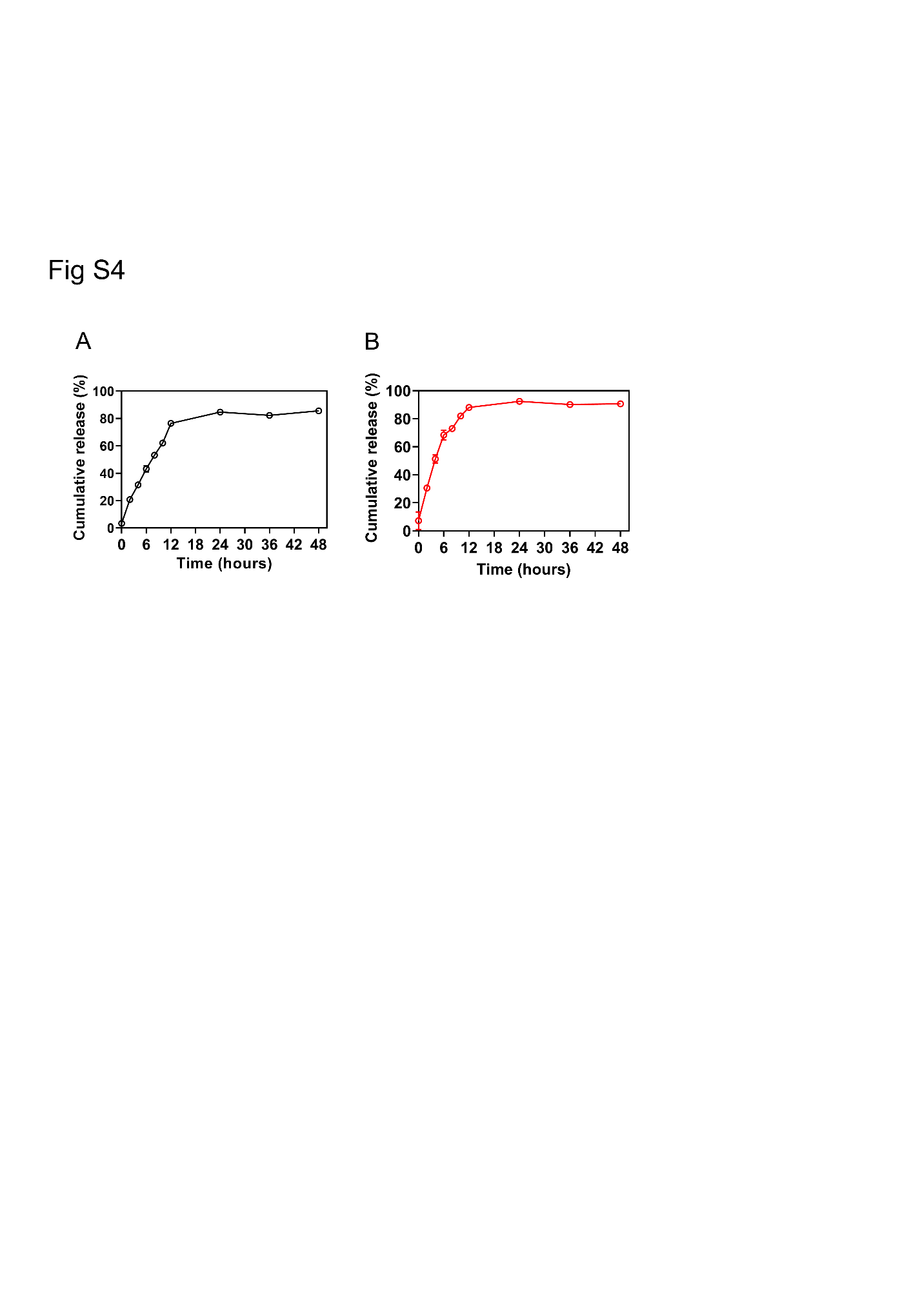


Figure S4. (A) In vitro cumulative release experiments of ropivacaine in an acidic environment (pH = 6.0), n = 3. (B) In vitro cumulative release experiments of cisplatin in an acidic environment (pH = 6.0), n = 3.

**Figure S5**


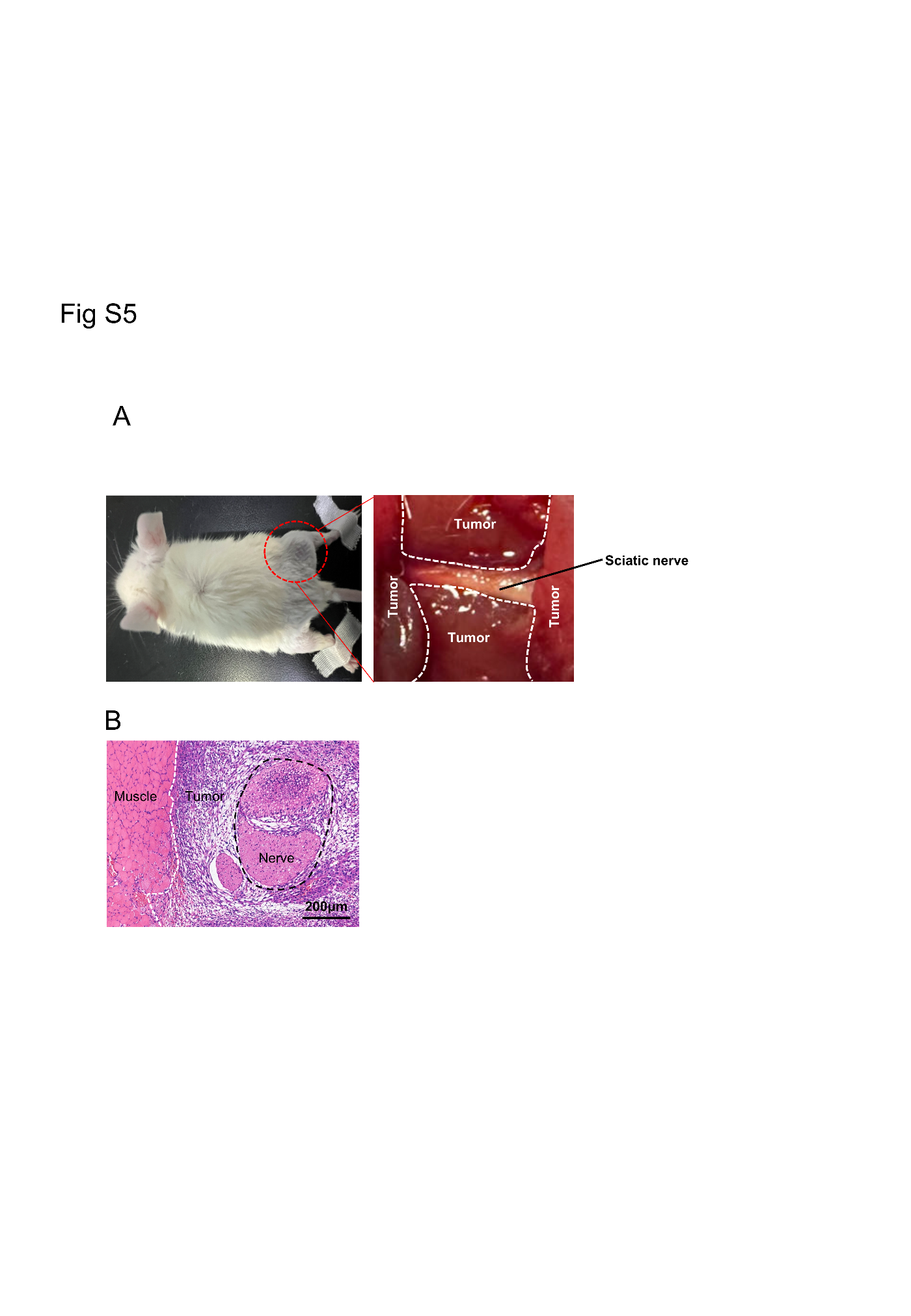


Figure S5. Anatomical location of tumor. (A) Anatomical location of tumor in mice. (B) H&E staining image of tumor side.

**Figure S6**


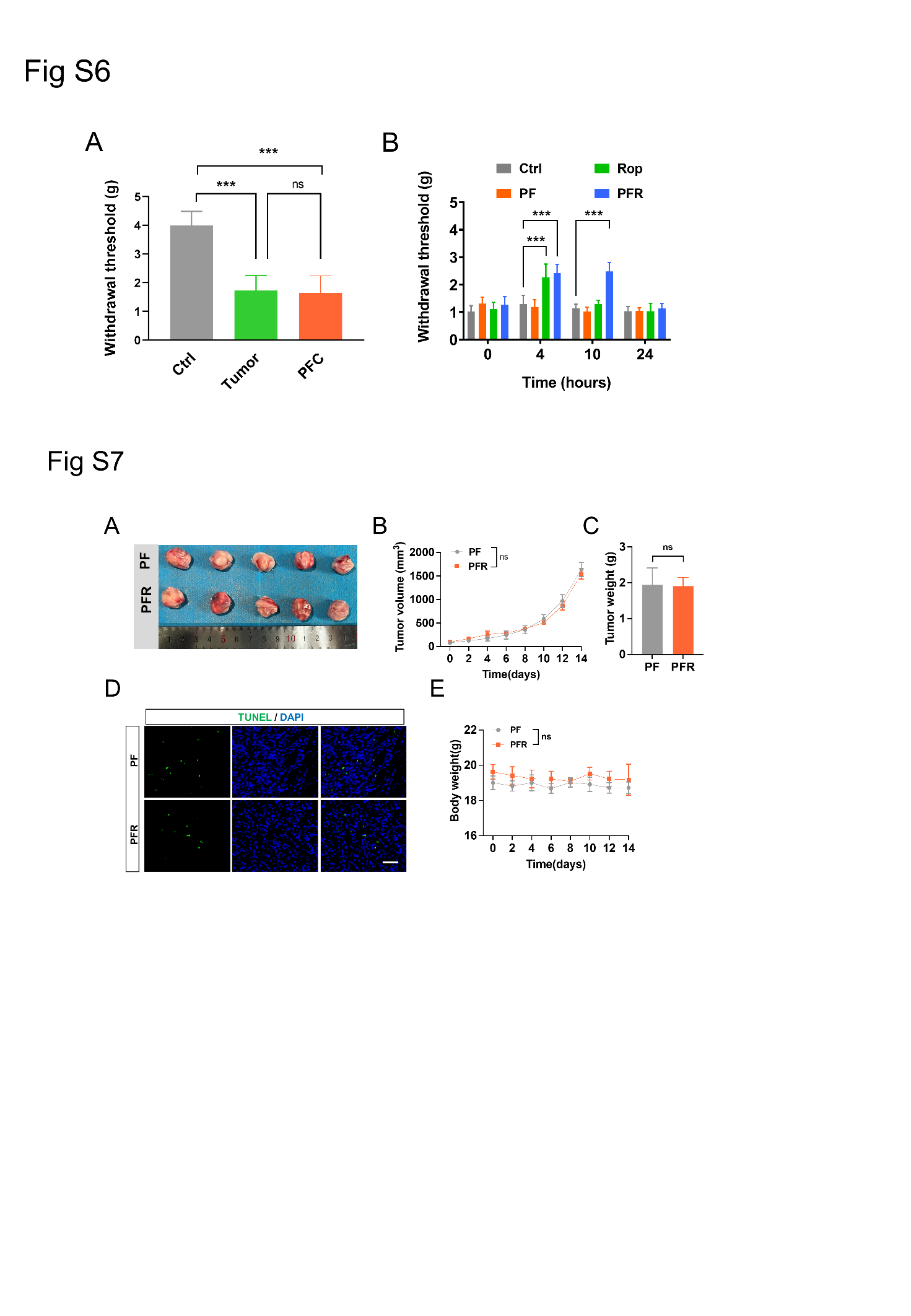


Figure S6. (A) Mechanical withdrawal threshold in tumor-free mice (Ctrl), tumor-inoculated mice and tumor-inoculated mice treated with PFC, n = 7. (B) Mechanical withdrawal threshold in tumor-inoculated mice was measured at 0, 4, 10, and 24 h after different treatments, n = 6. Ctrl: Saline; PF: PF127 hydrogel; Rop: ropivacaine; PFR: ropivacaine loaded PF127 hydrogel.

**Figure S7**


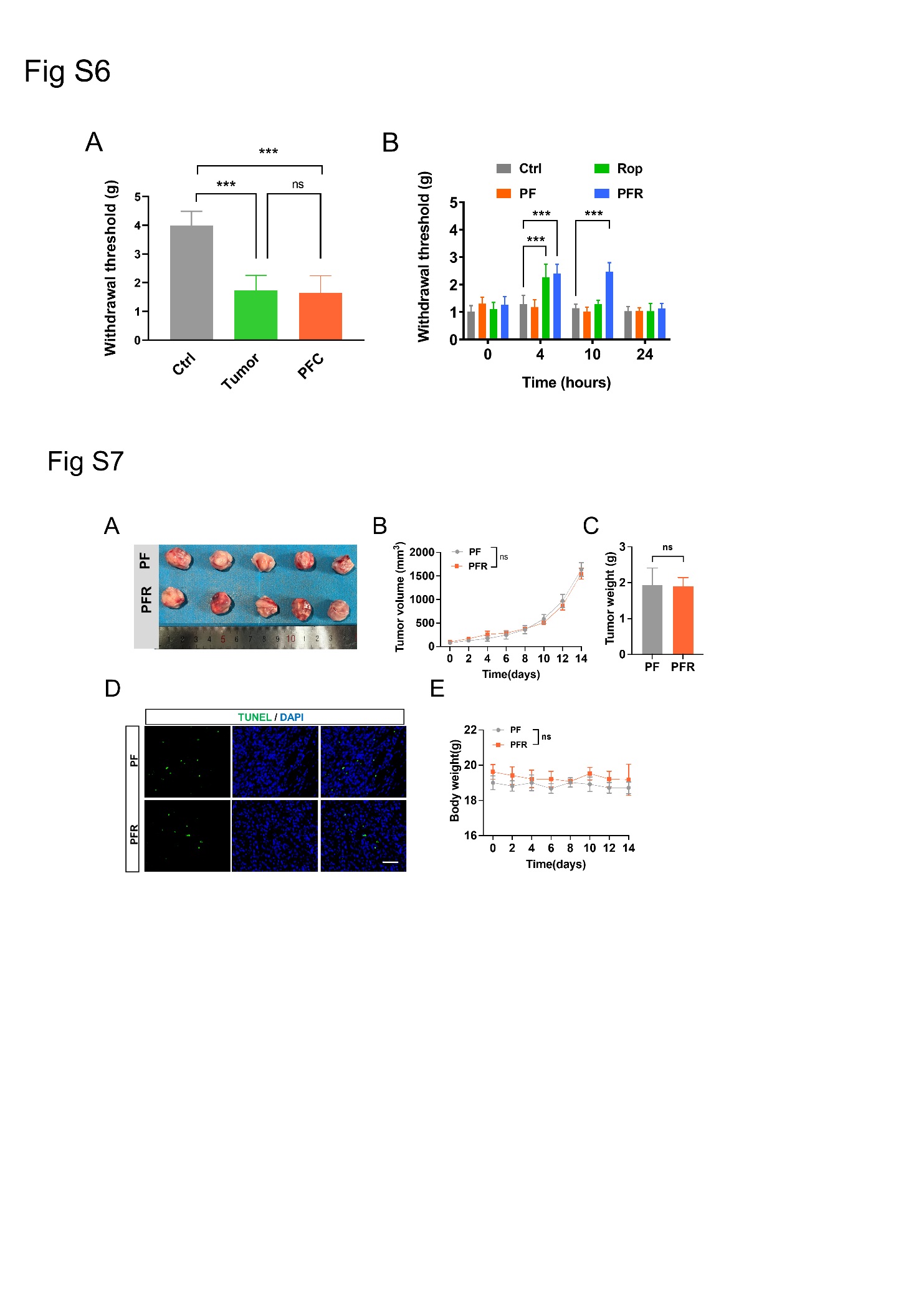


Figure S7. Anti-tumor effect of ropivacaine-loaded PF127 hydrogel. (A) Tumors after 14 days of different treatments. (B) Average tumor growth curves for 14 days, n = 5. (C) The weight of tumors on the 14th day, n = 5. (D) TUNEL staining for tumor sections was performed to identify apoptotic cells, while DAPI was used to stain the nuclei. Scale bar = 50 μm. (E) Body weight changes during the 14 days of treatments, n = 5.

**Figure S8**


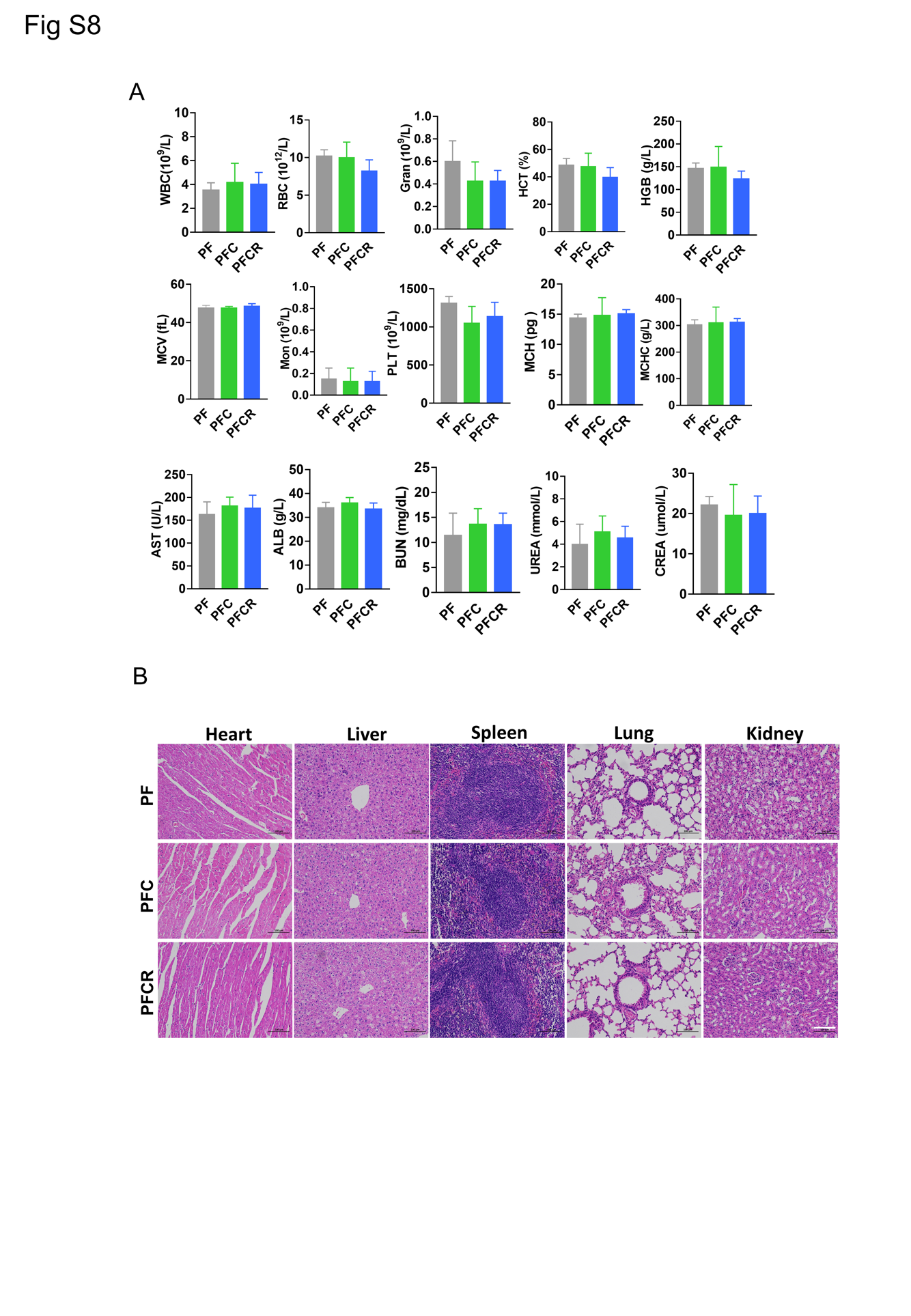


Figure S8. Assessment of systemic toxicity of PF127 hydrogel loaded with cisplatin and ropivacaine (PFCR) in mice. (A) The serum levels of WBC, RBC, Gran, HCT, HGB, MCV, Mon, PLT, MCH, MCHC, AST, ALB, BUN, UREA, CREA in mice teated with different hydrogels for 14 days, n = 4. (B) H&E staining images of the main organs of in mice teated with different hydrogels for 14 days, scale bar = 1mm.
